# Indications for the evaluation and supplementation of hypophosphatemia: an umbrella systematic review of reviews and guidelines

**DOI:** 10.1186/s12916-025-04415-1

**Published:** 2025-10-27

**Authors:** Seraina Netzer, Lea Büchel, Annina E. Büchi, Carole E. Aubert

**Affiliations:** 1 Department of General Internal Medicine, Inselspital, Bern University Hospital, University of Bern, Bern, Switzerland; 2 Faculty of Medicine, University of Bern, Bern, Switzerland; 3 Department of Pneumology, Inselspital, Bern University Hospital, University of Bern, Bern, Switzerland; 4 Institute of Primary Health Care (BIHAM), University of Bern, Bern, Switzerland

**Keywords:** Hypophosphatemia, Phosphate supplementation, Umbrella review

## Abstract

**Background:**

Hypophosphatemia, defined as low serum phosphate levels, is a frequent yet underrecognized condition associated with significant morbidity. Its etiology ranges from chronic conditions such as osteomalacia to acute states such as refeeding syndrome. This review systematically summarizes evidence and guidelines for phosphate testing and supplementation in adults, aiming to support clinical decision-making.

**Methods:**

We conducted a systematic review following the PRISMA guidelines. Searches of MEDLINE, Embase, the Cochrane Library, and Google Scholar from inception to September 2024 included reviews, guidelines, and consensus statements addressing phosphate measurement for hypophosphatemia and supplementation in adults outside intensive care settings. Eligibility criteria included English-language publications focused on diagnostic and therapeutic recommendations. Quality assessment was performed using the AGREE II tool, and data were synthesized across chronic and acute clinical contexts.

**Results:**

Thirty-three publications (11 guidelines, 19 reviews, and 3 consensus statements) were included, with high heterogeneity in the recommendations. Phosphate measurement to evaluate chronic hypophosphatemia is recommended for persistent musculoskeletal symptoms, osteoporosis evaluation, and rare conditions known to cause chronic hypophosphatemia, such as X-linked hypophosphatemia and tumor-induced osteomalacia. The post-kidney transplantation stage requires intensive early monitoring for hypophosphatemia. Recommendations for testing for drug-induced hypophosphatemia, such as with ferric carboxymaltose, vary. Phosphate measurement to evaluate acute hypophosphatemia is advised in high-risk settings: refeeding syndrome, hyperglycemic hyperosmolar syndrome, alcoholic ketoacidosis, worsening COPD or asthma exacerbations. Further potential indications for phosphate measurement include certain iron infusions, tenofovir treatment, immediate post-kidney transplantation, and intensive hemodialysis. Supplementation is indicated for severe or symptomatic cases, with oral therapy preferred for chronic conditions and intravenous routes for acute, severe hypophosphatemia.

**Conclusions:**

The heterogeneity in the recommendations emphasizes the need for individualized approaches based on clinical context. While robust evidence supports testing and supplementation under select conditions, gaps remain regarding optimal dosing and monitoring protocols. Clinicians should consider phosphate testing in high-risk scenarios and follow evidence-based supplementation guidelines tailored to chronic and acute hypophosphatemia. Future research is needed to unify recommendations and address existing uncertainties.

**Supplementary Information:**

The online version contains supplementary material available at 10.1186/s12916-025-04415-1.

## Background

Phosphate, an essential mineral in the human body, plays a pivotal role in various physiological processes, such as the regulation of acid‒base balance and bone health [1, 2]. Phosphate levels below normal, defined as hypophosphatemia, affect 5 to 30% of adults, depending on the setting and population [1]. There is no universal consensus on laboratory thresholds for hypophosphatemia; however, it is generally defined as a serum phosphate concentration below 0.8 mmol/L (≈2.5 mg/dL). Moderate hypophosphatemia is usually considered between 0.32–0.64 mmol/L (≈1.0–2 mg/dL), while severe hypophosphatemia is defined as < 0.32 mmol/L (≈1.0 mg/dL) [3]. Hypophosphatemia can result from inadequate dietary intake, excessive loss through the gastrointestinal tract or kidneys, or shifts of phosphate into cells, as observed in refeeding syndrome or acute respiratory alkalosis [4, 5]. Excessive loss can occur due to elevated levels of fibroblast growth factor 23 (FGF-23), which impairs phosphate reabsorption in the kidneys and reduces phosphate absorption in the intestines, as observed in conditions such as X-linked hypophosphatemia and tumor-induced osteomalacia [6]. Additionally, certain medications, such as certain iron infusions or chemotherapeutic drugs, may contribute to phosphate depletion [7]. In case of iron infusions, this also seems to be caused by high FGF-23 [8].

Hypophosphatemia can be acute and life threatening or chronic and can lead to impaired quality of life through symptoms such as muscle weakness and bone pain [9, 10]. Timely diagnosis and intervention are therefore crucial. This should address not only hypophosphatemia, but also the underlying cause. However, we have found no review summarizing all indications for testing for and supplementing hypophosphatemia. This literature review aims to fill this void and summarize the current evidence and guidelines for diagnosing and supplementing hypophosphatemia in adults, providing valuable insights for healthcare providers.

## Methods

We conducted a systematic literature review in accordance with the PRISMA statement [11].

### Information sources and search strategy

We conducted a search of MEDLINE, Embase, the Cochrane Library, and Google Scholar until September 2024, using any combination of the keywords “phosphate,” “phosphorus,” “screen*,” “test*,” “supplement*,” “substitu*,” “hospital*,” “inpatients,” “patient care,” “internal medicine,” “primary care,” and “ambul*”. The search strategy was designed via a text analysis of key papers. An animal study exclusion search filter was applied and adapted. For the detailed search strategy, see Additional file 1. Additionally, we searched reference lists and performed internet searches for potentially relevant guidelines not referenced, including the National Institute of Health and Care Excellence (NICE) website.

### Eligibility criteria

Reviews, guidelines, and consensus statements concerning when to test for hypophosphatemia and/or when to supplement phosphate in adults aged 18 years and older in any health care setting except intensive care units (ICUs) were included. ICU settings were excluded due to their highly specific clinical context, which differs substantially from general inpatient and outpatient care and often involves distinct protocols for phosphate management. Conference abstracts, books, study protocols, and publications in languages other than English were excluded. We excluded non-English publications to ensure consistent interpretation and quality assessment across sources, as translation may introduce bias or misinterpretation. Only the most recent publication was included, with the following exceptions: if an older publication had more or other information, then both publications were included to avoid losing relevant information. If a more recent publication had less information than an older publication and no new aspects, the older publication was retained.

We had initially planned to consider any type of study that assessed phosphate testing or supplementation and its appropriateness in adults. After the first 500 abstracts were reviewed, the inclusion rate was 26.3%. In a team discussion, we decided on the aforementioned selection criteria changes to include only reviews, guidelines, and consensus statements. The study protocol and amendments were preregistered in PROSPERO. Abstracts were screened independently by two reviewers (SN and LB or AEB), as were the full texts (SN and LB). Any disagreements were resolved by consensus or through discussion with the senior author (CEA).

### Data extraction

Data from all included publications were extracted independently by two reviewers (SN and LB), and disagreements were resolved by discussion. The following data items were extracted: authors, year of publication, publication type, number of involved specialties, involved countries, whether acute or chronic hypophosphatemia was addressed, when to test for hypophosphatemia, when to supplement, how to supplement, and difficulties or side effects of phosphate supplementation.

### Quality assessment

To assess the quality of the publications, we used the Appraisal of Guidelines, Research and Evaluation (AGREE) II tool [12]. Two researchers (SN and AEB) independently assessed the article quality. Each domain is assessed from 1 (strongly disagree) to 7 (strongly agree). We defined the risk of bias as high when the score was 1–2 points, moderate when the score was 3–5 points, and low when the score was 6–7 points.

### Data analysis

The findings were categorized on the basis of indications for phosphate testing and supplementation, separated into chronic and acute hypophosphatemia owing to different clinical presentations, and further organized to align with clinical settings and specific conditions, as outlined in Tables 1, 2, and 3. We reported how to supplement it if it was addressed. We further summarized the difficulties and side effects of phosphate supplementation when this was addressed in the publication.

## Results

### Study selection and characteristics

**Fig. 1:**
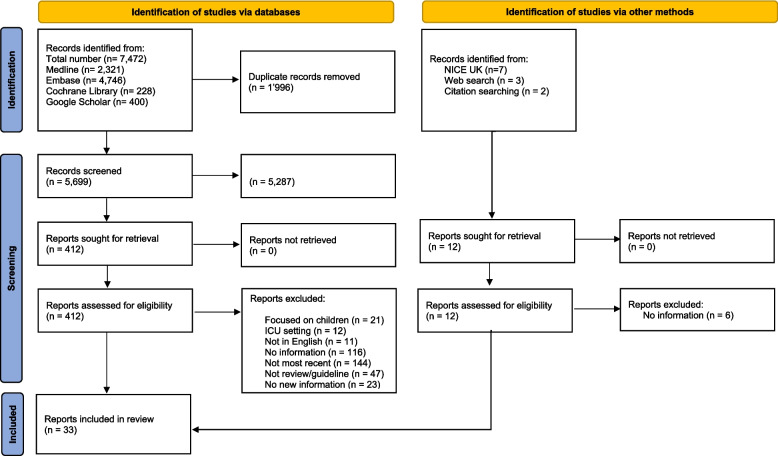
Flowchart showing the flow of articles through the identification, screening, and review process according to the Preferred Reporting Items for Systematic Reviews and Meta-Analyses Statement template

After removing duplicate records, 5,699 records were screened, 412 were assessed for eligibility, and 33 were included in this review, comprising 11 guidelines [4, 13–22], 19 reviews [8, 23–40], and 3 consensus statements [9, 41, 42] (Fig. 1).

### Quality assessment

Study quality was very mixed, ranging from the highest quality guidelines [16, 19] to expert opinions without a systematic literature search [9, 24, 28], as detailed in Additional file 1: Table S1.

### Chronic hypophosphatemia: testing

Chronic hypophosphatemia was defined as serum phosphate levels below the normal range of 0.81–1.45 mmol/L (2.5–4.5 mg/dL) on at least three consecutive fasting morning measurements taken 3 months apart in one included article (Table 1) [24]. Testing for chronic hypophosphatemia is recommended in morning fasting blood samples and in cases of persistent musculoskeletal symptoms; clinical scenarios where phosphate evaluation is indicated include suspected osteomalacia, which is based on symptoms such as bone pain, muscle weakness, and pseudofractures [23, 43], as well as during evaluations for osteoporosis or fragility fractures [17]. Tumor-induced osteomalacia also warrants testing in unexplained musculoskeletal symptoms or abnormal DXA scans [14]. For X-linked hypophosphatemia, a genetic condition most commonly diagnosed in childhood due to rickets, annual monitoring is advised for untreated adult patients, and semiannual monitoring is advised for those on treatment [13], with first monitoring after the initiation of supplementation at 3 months [41] or monthly monitoring until stable [26]. Treatment of X-linked hypophosphatemia with burosumab should also be monitored. Fibrous dysplasia/McCune–Albright syndrome necessitates testing during disease staging or when skeletal symptoms change [15]. The updated international guidelines on the evaluation and management of primary hyperparathyroidism state that measuring phosphate when evaluating the condition is not essential but can be helpful and is recommended [18]. Japanese experts recommend testing phosphate as the first step in evaluating the cause of hypocalcemia [42]. Most kidney transplant patients experience hypophosphatemia within the first 3 months after transplantation due to renal phosphate loss [37]. Weekly phosphate testing is thus recommended during this period until levels stabilize, followed by monitoring every 6 to 12 months during later stages of chronic kidney disease [16].

**Table 1:** Results part 1: When to test for hypophosphatemia

Paper (author year)	Cause/symptom	When to test
***Chronic hypophosphatemia***		
Fukumoto 2015 [9] Consensus statement	Osteomalacia	• In suspected osteomalacia: bone pain, muscle weakness, pigeon chest, spinal curvature, pseudofractures
Ackah 2022 [23] Review	Hypophosphatemic osteomalacia	• To monitor treatment every 3–4 months, in pregnancy at least monthly
Gregson 2022 [17] Guideline	Osteoporosis	• Investigation of osteoporosis or fragility fractures
Jan de Beur 2023 [14] Guideline	Tumor-induced osteomalacia	• In chronic muscle pain, weakness, fragility fractures, and bone pain • Monitor stable doses every 3–4 months • Monitor frequently after surgery until normalization, then annually
Haffner 2019 [13] Guideline	X-linked hypophosphatemia	• Monitor once a year if not treated
Trombetti 2022 [41] Consensus statement	X-linked hypophosphatemia	• Monitor 3 months after treatment initiation, every 6 months in stable condition • Monitor pregnant and lactating people • Burosumab treatment: every 2 weeks after treatment initiation, every 4 weeks for the following 2 months, 4 weeks after dose adjustment (then as appropriate)
Cherian 2024 [26] Review	X-linked hypophosphatemia	• Under treatment monitor monthly until stable, then every 3–4 months
Javaid 2019 [15] Guideline	Fibrous dysplasia/McCune–Albright syndrome	• When staging, in all with suspected polyostotic disease, change of skeletal symptoms
Aljuraibah 2022 [24] Review	Chronic hypophosphatemia	• Definition of chronic hypophosphatemia: 3 consecutive morning fasting measurements at least 3 months apart
Bilezikian 2022 [18] Guideline	Primary hyperparathyroidism	• When evaluating patients with primary hyperparathyroidism
Fukumoto 2008 [42] Consensus statement	Hypocalcemia	• When evaluating the cause of hypocalcemia
KDIGO 2017 [16] Guideline	Kidney transplant	• Weekly during immediate post-transplantation stage until stable • Every 6–12 months in CKD stages 1–3
***Acute hypophosphatemia***		
ASPEN 2020 [4] Guideline	Refeeding	• Risk evaluation for refeeding in populations potentially at risk* • Before initiation of nutrition, every 12 h for 3 days in high risk^Ω^
Reber 2019 [34] Review	Refeeding in medical inpatients	• Nutrition initiation: day 1–3 monitor daily, day 4–6 every second day, day 7–10 1–2 × /week • Monitor supplementation: next day in mild (0.61–0.8 mmol/L; 1.9–2.5 mg/dL), after 8–12 h in moderate (≤ 0.6 mmol/L; ≈1.9 mg/dL) hypophosphatemia
Kraft 2015 [29] Review	Nutrition support, refeeding	• Nutrition initiation: daily in well-nourished, every 8–12 h for the first several days in malnourished • Monitor i.v. supplementation: after 2–24 h
NICE 2017 [19] Guideline	Refeeding	• Nutrition initiation^∞^: baseline, daily if at risk of refeeding, 3 × /week until stable, then weekly
Milionis 2005 [32] Review	Hyperglycemic hyperosmolar syndrome	• All with HHS: baseline, every 2–6 h during initial treatment
Long [31] Review	Alcoholic ketoacidosis	• Monitor during alcoholic ketoacidosis treatment
Miller 2000 [33] Review	COPD/asthma in the emergency department	• In COPD or asthma exacerbations that do not improve/worsen
Nesrallah 2013 [20] Guideline	Intensive hemodialysis**	• Predialysis
***Drug-associated hypophosphatemia***		
Boots 2022 [8] Review/pragmatic guideline	Iron infusion	• Before infusion, 1–2 weeks after FCM • Monitor supplementation
Schaefer 2022 [36] Review	Iron infusion	• No universal screening • Ongoing fatigue, bone pain, muscle weakness after infusion • Before repeat FCM infusions
Vilaca 2022 [38] Systematic review	Iron infusion–osteomalacia	• In repeated iron infusions, at 2 and 5 weeks after infusion
Rodriguez-Pascual 2010 [35] Review	mTOR-Inhibitors	• No information on initial testing • Monitor monthly in mild, weekly in moderate, and daily in severe hypophosphatemia
Lampertico 2016 [30] Review	TDF in HBV-infection	• Before initiation of treatment with TDF • During treatment every 3 months for 1 year, then every 6 months • High risk every month for 3 months, then as above • Close monitoring in worsening kidney function
Eacs 2023 [21] Guideline	HIV–TDF	• In patients with TDF and worsening eGFR or proteinuria
OARAC 2024 [22] Guideline	HIV–TDF	• Every 6–12 months in patients with CKD and TDF

*HHS *hyperglycemic hyperosmolar syndrome, *FCM *ferric carboxymaltose, *TDF *tenofovir disoproxil fumarate, *HBV *hepatitis B virus, *eGFR *estimated glomerular filtration rate, *CKD *chronic kidney disease

^*^Population potentially at risk for refeeding, e.g., anorexia nervosa, alcohol disorder, bariatric/bowel resection, famine, malignancy

^Ω^High risk patients for refeeding syndrome: BMI < 16 kg/m^2^, weight loss of 7.5% in the last 3 months or 10% in the last 6 months, < 50% of oral intake for the last 5 + days, severe disease, and low phosphate before nutrition initiation

^∞^Consider nutritional support in malnourished patients (BMI < 18.5; unintentional weight loss > 10% in 3–6 months; BMI < 20 and weight loss > 5% in 3–6 months) and monitoring during pregnancy

^**^ Intensive hemodialysis was defined as short daily (≥ 3.0 h, 5–7 days per week) or long (often nocturnal; ≥ 5.5 h, ≥ 3 sessions per week)

### Chronic hypophosphatemia: supplementation

Phosphate supplementation is generally recommended for symptomatic chronic hypophosphatemia; common conditions requiring supplementation include osteomalacia, where recommendations for oral supplementation range from 20–60 mg (≈0.65–1.93 mmol)/kg/day to 750–2000 mg (≈24.2–64.5 mmol)/day in 2–5 doses until clinical improvement [23, 27]. In X-linked hypophosphatemia, supplementation is indicated in cases of musculoskeletal pain, pseudofractures, or planned dental or orthopedic surgery, with the lowest dose consistent with the relief of symptoms, ranging from 750 to 2000 mg (≈24.2–64.5 mmol)/day, as well as during pregnancy [13, 41]. For tumor-induced osteomalacia, supplementation should be followed promptly at diagnosis until tumor removal and should be continued if a surgical cure is not possible. For fibrous dysplasia/McCune–Albright syndrome, supplementation is suggested when serum phosphate levels are below normal, with doses ranging from 155 to 300 mg/day and titrated to the lower norm of phosphate levels [15]. The supplementation recommendation thresholds in the post-kidney transplant stage vary from < 0.65 mmol/L (2.0 mg/dL) to < 1.0 mmol/L (3.1 mg/dL) if muscle weakness is present [25, 37].

### Acute hypophosphatemia: testing

Acute hypophosphatemia can be critical in high-risk clinical scenarios, particularly when shifts in phosphate levels occur due to underlying conditions. A well-known cause of acute hypophosphatemia is refeeding syndrome, which necessitates routine phosphate monitoring before and during nutrition initiation, with differing monitoring intervals depending on baseline risk and phosphate levels [4, 19, 29, 34]. Further clinical scenarios that might warrant phosphate testing include conditions where treatment commonly leads to an intracellular shift—diabetic ketoacidosis [28], hyperglycemic hyperosmolar syndrome [32], or alcoholic ketoacidosis [31]—as well as COPD or asthma exacerbations that do not improve with standard therapy [33]. Hypophosphatemia can occur in end-stage CKD when patients undergo intensive dialysis, which is defined as short daily (< 3 h, 5–7 days/week) or long (often nocturnal, ≥ 5.5 h, ≥ 3/week) sessions. In this situation, it is recommended to measure phosphate before dialysis and to use enriched dialysate (0.65–2.6 mmol/L; ≈2–8 mg/dL) [40] with the goal of normal phosphate levels predialysis [20].

### Acute hypophosphatemia: supplementation

Supplementation in refeeding syndrome patients is warranted with nutrition initiation if the phosphate concentration is < 0.8 mmol/L (2.5 mg/dL) before nutrition initiation and prophylactically in very high-risk patients (BMI < 14 kg/m^2^, starvation > 15 days, weight loss > 20% in the last 3–6 months) [34] and in all receiving enteral or parenteral nutrition [29]. Dose recommendations vary (Table 3). For other acute hypophosphatemia causes, supplementation is generally warranted for severe, as well as moderate but symptomatic, hypophosphatemia [39]. Oral supplementation can be considered for moderate hypophosphatemia, whereas the intravenous route is preferred for severe hypophosphatemia, with varying dose recommendations (Tables 2 and 3).

**Table 2:** Results part 2: When to supplement hypophosphatemia

Paper (author year)	Cause/symptom	When to supplement
***Chronic hypophosphatemia***		
Ackah 2022 [23] Review	Hypophosphatemic osteomalacia	• All with symptomatic hypophosphatemic osteomalacia
Jan de Beur 2023 [14] Guideline	Tumor-induced osteomalacia	• Promptly when TIO is biochemically confirmed until surgical removal • Continue when surgical cure is not possible
Haffner 2019 [13] Guideline	X-linked hypophosphatemia	• Symptomatic (musculoskeletal pain, pseudofractures, dental issues), planned orthopedic or dental surgery, biochemical evidence of osteomalacia' • Planned dental implant surgery: start 3–6 months before and continue 6 months after surgery • Pregnancy: consider and continue until end of breastfeeding
Trombetti 2022 [41] Consensus statement	X-linked hypophosphatemia	• Symptomatic (bone pain, pseudofractures) • Planned dental or orthopedic surgery: start before and continue 3–6 months after surgery • Pregnancy: consider and continue until end of breastfeeding
Javaid 2019 [15] Guideline	Fibrous dysplasia/McCune–Albright syndrome	• Serum phosphate levels below normal
Vangala 2018 [37] Review	Kidney transplant	• Phosphate < 0.65 mmol/L (< 2 mg/dL)
Baia 2015 [25] Review	Kidney transplant	• Phosphate < 0.5 mmol/L (1.55 mg/dL) or < 1 mmol/L (3.1 mg/dL) and muscle weakness
***Acute hypophosphatemia***		
Reber 2019 [34] Review	Refeeding in medical inpatients	• With nutrition initiation when phosphate < 0.8 mmol/L (< 2.5 mg/dL), prophylactic in very high risk (BMI < 14 kg/m^2^, starvation > 15 days, weight loss > 20% in the last 3–6 months)
Kraft 2015 [29] Review	Nutrition support, refeeding	• All receiving enteral or parenteral nutrition
Gosmanov 2014 [28] Review	Diabetic ketoacidosis	• When phosphate < 0.32 mmol/L (< 1 mg/dL), or < 0.64 mmol/L (< 2 mg/dL) and cardiac dysfunction, anemia or respiratory depression
Milionis 2005 [32] Review	Hyperglycemic hyperosmolar syndrome	• When phosphate < 0.32 mmol/L (< 1 mg/dL)
Long [31] Review	Alcoholic ketoacidosis	• When phosphate < 0.65 mmol/L (< 2 mg/dL)
Weber 2019 [39] Review	Severe hypophosphatemia	• Severe hypophosphatemia (< 0.32 mmol/L (< 1 mg/dL)) • Mild or moderate when symptomatic^µ^
Miller 2000 [33] Review	COPD/asthma in the emergency department	• Phosphate < 0.32 mmol/L (< 1 mg/dL) • Low phosphate and significant symptoms^α^
***Drug-associated hypophosphatemia***		
Boots 2022 [8] Review/pragmatic guideline	Iron infusion	• Consider in moderate (< 0.65 mmol/L; 2 mg/DL) or severe (< 0.35 mmol/L; ≈1 mg/dL) hypophosphatemia
Rodriguez-Pascual 2010 [35] Review	mTOR-Inhibitors	• Phosphate < 0.81 mmol/L (< 2.5 mg/dL)

*TIO *tumor-induced osteomalacia

Biochemical evidence of osteomalacia: increase in serum levels of bone-specific ALP

^µ^Symptoms include decreases in cardiac and respiratory muscle function, intestinal ileus, mental status changes, and rhabdomyolysis

^α^Significant symptoms include altered mental status, hypotension, arrhythmia, and respiratory distress

**Table 3:** Results part 3: How to supplement hypophosphatemia

Paper (author year)	Cause/symptom	How to supplement
***Chronic hypophosphatemia***		
Ackah 2022 [23] Review	Hypophosphatemic osteomalacia	• Orally 20–60 mg/kg/day in 3–5 doses until clinical improvement • Decrease dose if phosphate is elevated
Haffner 2019 [13] Guideline	X-linked hypophosphatemia	• 750–1600 mg/day in 2–4 divided doses, increased gradually • Stop in patients with markedly increased parathyroid hormone levels • During pregnancy up to 2′000 mg/day
Trombetti 2022 [41] Consensus statement	X-linked hypophosphatemia	• 0–2000 mg/day, lowest dose consistent with relief of symptoms
Javaid 2019 [15] Guideline	Fibrous dysplasia/McCune–Albright syndrome	• 155–300 mg/day, titrated to lower end or just below of normal
Florenzano 2020 [27] Review	Hypophosphatemia overall	• Osteomalacia: 0.75–1 g/day in 3–4 doses, or 0–2 g/day in 2 doses; pregnancy 2 g/day in 2 doses; menopause 0–2 g/day in 2 doses • Intravenous in acute symptomatic, oral in other (chronic or acute asymptomatic)
Vangala 2018 [37] Review	Kidney transplant	• Orally until phosphate 0.65 mmol/L (2 mg/dL)
***Acute hypophosphatemia***		
Reber 2019 [34] Review	Refeeding in medical inpatients	• Mild (0.61–0.8 mmol/L; 2–2.5 mg/dL) 0.3 mmol (9.3 mg)/kg/day orally in divided doses or i.v. over 8–12 h • Moderate (0.32–0.6 mmol/L 1–2 mg/dL) i.v. 0.6 mmol/kg/day over 8–12 h • Devere (< 0.32 mmol/L; 1 mg/dL) same as moderate
Kraft 2015 [29] Review	Nutrition support, refeeding	• Enteral nutrition contains 700–1200 mg/L • Mild (0.75–0.9 mmol/L; 2.3–2.8 mg/dL) i.v. 0.08–0.16 mmol/kg over 4–6 h • Moderate (0.5–0.75 mmol/L; 1.55–3.2 mg/dL) i.v. 0.16–0.32 mmol/kg over 4–6 h • Severe (< 0.5 mmol/L; < 1.55 mg/dL) i.v. 0.32–0.64 mmol/kg over 4–6 h. Max. 7–7.5 mmol/h, in decreased GFR consider halving dose, consider using adjusted body weight
NICE 2017 [19] Guideline	Refeeding	• Likely requirement 0.3–0.6 mmol/kg/day
Gosmanov 2014 [28] Review	Diabetic ketoacidosis	• i.v. 0.1–0.2 mmol/kg over 6 h
Milionis 2005 [32] Review	Hyperglycemic hyperosmolar syndrome	• i.v. < 3–4 mmol/h in a 60–70 kg person
Long [31] Review	Alcoholic ketoacidosis	• If 0.32–0.65 mmol/L(1–2 mg/dL) oral potassium-phosphate 250–500 mg (8–16.1 mmol) every 12 h or 15 mmol i.v. over 2.5 h • If < 0.32 mmol/L potassium-phosphate 45 mmol i.v. over 7 h
Weber 2019 [39] Review	Severe hypophosphatemia	• If > 0.4 mmol/L (1.24 mg/dL) i.v. 0.08–0.24 mmol/kg over 6 h; max 30 mmol • If < 0.4 mmol/L (1.24 mg/dL) i.v. 0.25–0.5 mmol/kg over 8–12 h max 80 mmol • Once 0.48 mmol/L (1.49 mg/dL) switch to oral 40–80 mmol(1240–2480 mg)/day in 3–4 doses
Miller 2000 [33] Review	COPD/asthma in the emergency department	• Non-life-threatening 30 mmol/day • Life-threatening 90 mmmol/day
Nesrallah 2013 [20] Guideline	Intensive hemodialysis	• Enriched dialysate with goal normal phosphate level predialysis
Sam 2006 [40] Review	Hemodialysate composition	• Phosphate enriched dialysate 0.65–2.6 mmol/L(≈2–8 mg/dL), most commonly 1.3 mmol/L(≈4 mg/dL)
***Drug-induced hypophosphatemia***		
Boots 2022 [8] Review/pragmatic guideline	Iron infusion	• Orally for moderate (< 0.65–0.35 mmol/L; ≈1–2 mg/dL) asymptomatic, i.v. for moderate symptomatic or severe (< 0.35 mmol/L; ≈1 mg/dL)
Rodriguez-Pascual 2010 [35] Review	mTOR-Inhibitors	• Mild (0.65–0.81 mmol/L; ≈2–2.5 mg/dL) and moderate (0.32–0.65 mmol/L; 1–2 mg/dL) orally 1–2 g/day, divided in 3–4 doses • Severe (< 0.32 mmol/L; < 1 mg/dL) i.v

*i.v* intravenous

250 mg of phosphate supplementation correspond to 8.06 mmol

### Drug-associated hypophosphatemia: testing

Ferric carboxymaltose (FCM) and tenofovir disoproxil fumarate (TDF) can cause hypophosphatemia. Testing recommendations for FCM-induced hypophosphatemia vary from before infusion and 1 to 5 weeks after infusion in patients undergoing repeated infusions [8, 38] to only symptomatic patients [36]. For TDF, recommendations vary from monitoring phosphate levels every 3 to 12 months during treatment [22, 30] or only in patients with declining renal function [21]. mTOR inhibitors are associated with hypophosphatemia, and monitoring is recommended monthly for mild hypophosphatemia, weekly for moderate hypophosphatemia, and daily for severe hypophosphatemia, with no recommendation for initial phosphate measurement [35].

### Drug-associated hypophosphatemia: supplementation

Supplementation for FCM-induced hypophosphatemia depends on severity, with oral doses for moderate hypophosphatemia and intravenous administration for severe hypophosphatemia [8]. Supplementation for mTOR inhibitor-induced hypophosphatemia is recommended when the phosphate level is < 0.81 mmol/L (2.5 mg/dl), with oral administration for mild and moderate hypophosphatemia and intravenous administration for severe hypophosphatemia (Table 3) [35].

### Adverse outcomes associated with phosphate testing and supplementation

We found no mention of adverse outcomes associated with testing. For adverse outcomes associated with supplementation, multiple reviews mentioned the risk of secondary or tertiary hyperparathyroidism, as well as renal issues such as nephrocalcinosis in cases of long-term oral supplementation (Table 4) [6, 23, 25, 27, 37, 44]. Management recommendations varied from laboratory monitoring to dose modification, the use of calcimimetics, surgical interventions, or the cessation of drugs that cause hypophosphatemia. Gastrointestinal symptoms such as diarrhea and dyspepsia were also commonly mentioned and recommended to be managed by careful dose upward titration [6, 23, 25, 27]. Due to rapid absorption and excretion, oral phosphate has to be given in several doses, which, in combination with the gastrointestinal effects, can lead to compliance issues [27]. For intravenous supplementation, hypocalcemia, thrombophlebitis, and arrhythmias were mentioned as adverse outcomes, and recommended to be managed with laboratory monitoring and low doses and infusion rate [8, 28, 29].

**Table 4:** Side effects of phosphate supplementation

Paper (author year)	Cause/symptom	Adverse outcome(s)	Management
Ackah 2022 [23]	Hypophosphatemic osteomalacia	• Secondary/tertiary hyperparathyroidism • Gastrointestinal symptoms	• Evaluation of calcimimetic, surgical intervention • Dose reduction and gradual upward titration
Jan de Beur 2023 [14]	Tumor-induced osteomalacia	• Secondary/tertiary hyperparathyroidism • Nephrolithiasis, nephrocalcinosis, impaired renal function • Gastrointestinal symptoms	• Avoid supplementation outside of bridging time between diagnosis and surgical removal
Haffner 2019 [13]	X-linked hypophosphatemia	• Secondary/tertiary hyperparathyroidism • Gastrointestinal symptoms	• Laboratory monitoring of PTH, stop supplementation if markedly increased • Gradual upward titration
Cherian 2024 [26]	X-linked hypophosphatemia	• Hypercalcemia, worsening renal function, nephrocalcinosis, high PTH	• Dose reduction • Renal ultrasound every 1–2 years
Florenzano 2020 [27]	Hypophosphatemia overall	• Gastrointestinal symptoms • Secondary/tertiary hyperparathyrodism, nephrocalcinosis, renal impairment • Compliance due to 3–4 × doses/day	• Dose modification • Dose modification
Kraft 2015 [29]	Nutrition support, refeeding	• Calcium-phosphate precipitation, thrombophlebitis with i.v. supplementation	• Infuse i.v. doses over several hours, no more than 7–7.5 mmol/h
Gosmanov 2014 [28]	Diabetic ketoacidosis	• Hypocalcemia with i.v. supplementation	• Low doses and rates, close monitoring of phosphate and calcium
Boots 2022 [8]	Iron infusions	• Hypocalcemia, arrhythmias, ectopic calcifications, nephropathy	• Avoid FCM in patients with high risk of developing severe hypophosphatemia
Vangala 2018 [37]	Kidney transplantation	• Hyperparathyroidism, nephrocalcinosis	• Goal phosphate of 0.65 mmol/L (2 mg/dL)
Baia 2015 [25]	Kidney transplantation	• Secondary hyperparathyroidism, nephrocalcinosis • Gastrointestinal effects	• Minimum dose of supplements, frequent monitoring

*PTH *parathyroid hormone, *i.v *intravenous

### Result summary

**Fig. 2:**
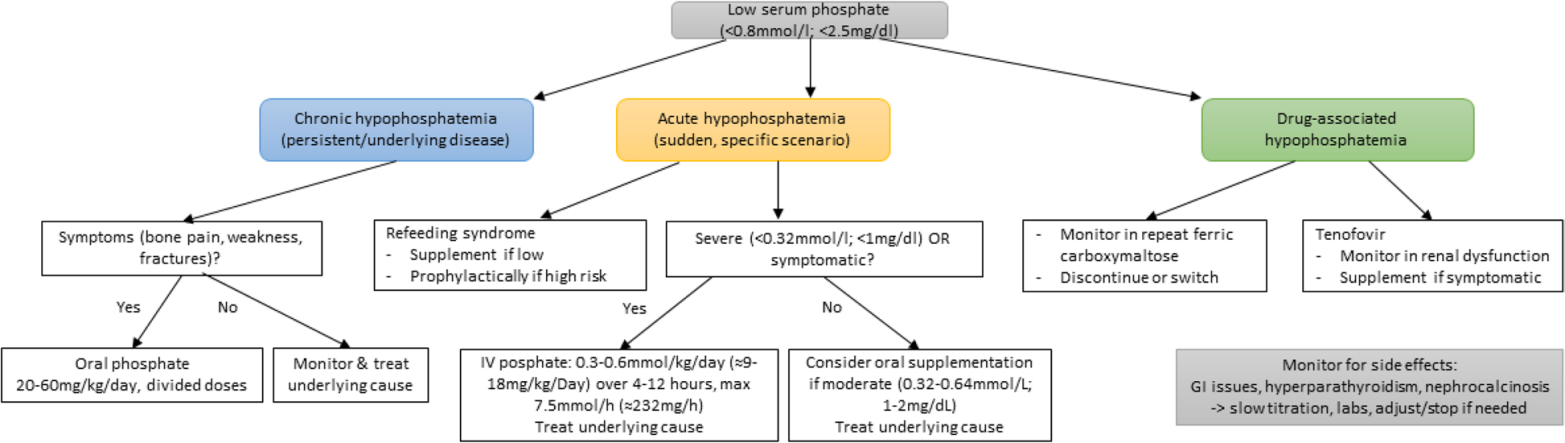
Proposed treatment algorithm for hypophosphatemia. This flowchart summarizes practical decision pathways for chronic, acute, and drug-induced hypophosphatemia. Oral supplementation is advised in symptomatic chronic cases, while severe or symptomatic acute cases require intravenous treatment. For details on refeeding syndrome, check Tables 1, 2, and 3. Drug-associated hypophosphatemia (notably ferric carboxymaltose, tenofovir) requires close monitoring, with supplementation guided by severity. Side effects (e.g., gastrointestinal intolerance, hyperparathyroidism, nephrocalcinosis) should be monitored and managed accordingly

A short overview on when to test for hypophosphatemia overall can be found in Table 5. Figure 2 summarizes the results of the review in an algorithm.

**Table 5:** When to test for hypophosphatemia

• Persistent musculoskeletal symptoms (bone pain, muscle weakness, fragility fractures, suspected osteomalacia) • Osteoporosis work-up or unexplained fractures • Consider when evaluating primary hyperparathyroidism or hypocalcemia • When reinitiating nutrition (refeeding syndrome) • Consider in high risk acute situations: Diabetic ketoacidosis Hyperglycemic hyperosomolar syndrome Alcoholic ketoacidosis COPD/asthma exacerbations without improvement • After kidney transplantation • Repeat ferric carboxymaltose infusions • Consider when treating with tenofovir (especially with renal dysfunction)	

Most common causes: For details and monitoring, check Table 1

## Discussion

In this systematic literature review, we identified a very heterogeneous data landscape with some well-researched topics, but overall, there is no clear consensus on when to test and when and how to supplement phosphate.

Chronic hypophosphatemia can lead to osteomalacia with debilitating symptoms and is often recognized late, which is why clinicians should be aware of this [6]. We support the recommendation of testing phosphate in patients with ongoing unclear musculoskeletal symptoms [9, 45]. It is also recommended as part of osteoporosis and insufficiency fracture evaluations [17, 46]. There are rare genetic disorders that cause hypophosphatemia, the most studied being X-linked hypophosphatemia, which is most commonly diagnosed in childhood and needs surveillance in adulthood [41]. It is important to note that phosphate levels can be affected by dietary intake and should best be measured as morning fasting phosphate [47]. If the cause of chronic hypophosphatemia cannot be eliminated (surgical removal in the case of tumor-induced osteomalacia or cessation of the causative drug, see below), supplementation is commonly recommended, always in symptomatic cases [23], Regimens vary with some dose suggestions considering body weight [23, 44], others with overall doses ranging from 150 to 2000 mg (4.8–64.5 mmol)/day [13, 15], but prospective studies evaluating the optimal doses are lacking. There is consensus on the need to divide the doses, which vary from 2 to 6/day, as phosphate is absorbed and excreted rapidly [27]. Treatment monitoring, if mentioned, is commonly recommended every 3–6 months, e.g., 3 months after treatment initiation and every 6 months when stable [41]. Treatment goals are not always defined but are most commonly clinical improvements, as laboratory targets do not lead to better symptom control in X-linked hypophosphatemia patients but potentially to more side effects [6, 13]. Common side effects of phosphate supplementation include gastrointestinal symptoms, which can be managed by dose reduction and slow upward titration [23, 27]. Long-term supplementation can further lead to secondary hyperparathyroidism and renal complications, with some authors recommending regular laboratory monitoring and/or lower laboratory or clinical targets, as mentioned above [23, 27, 44]. A new alternative treatment for FGF-23-mediated hypophosphatemia is the antibody burosumab, which is outside the scope of this review [41]. Much more common causes of hypophosphatemia are primary hyperparathyroidism, where phosphate testing is not strictly necessary but is recommended [18], and vitamin D deficiency, where phosphate testing and supplementation are seen as unnecessary [48, 49].

In summary, chronic hypophosphatemia should be sought in patients with unclear persistent musculoskeletal symptoms and should most likely be treated orally in several doses, starting with a low dose and slowly titrating upward until clinical improvement, while monitoring phosphate and signs of complications.

Acute hypophosphatemia can be caused by an intracellular shift, such as with hyperventilation or treatment of ketoacidosis with insulin [28, 31, 32, 50]. These shifts correct themselves and do not usually require treatment. Several studies on diabetic ketoacidosis have failed to show a benefit of phosphate supplementation [51, 52]; however, no such studies exist in other states, such as hyperglycemic hyperosmolar syndrome or alcoholic ketoacidosis. Nevertheless, some authors recommend phosphate testing during treatment of the aforementioned states to detect and supplement severe (< 0.32 mmol/L; < 1 mg/dL) and/or symptomatic hypophosphatemia [28, 31, 32]. A further publication recommended testing phosphate in patients with COPD or asthma due to potential hyperventilation causing a phosphate shift, especially when patients present in an exacerbated state that fails to improve despite adequate therapy [33]. It is also well known that hypophosphatemia is common in ventilated patients and is associated with difficulties in weaning [53], but as we decided to exclude the ICU setting, this is outside the scope of this review. Overall, in cases of severe hypophosphatemia (< 0.32 mmol/L; < 1 mg/dL) or moderate symptomatic (e.g., altered mental status, rhabdomyolysis, arrhythmia, respiratory distress), supplementation is commonly recommended, with intravenous doses of 0.3–0.6 mmol (9–18 mg)/kg/day administered over 4–12 h, with laboratory monitoring after 6–24 h [39, 54], although these recommendations mostly rely on case reports and expert opinions. The potential side effects of intravenous supplementation are hypocalcemia and arrhythmias; thus, infusions over several hours with a maximum infusion rate of 7.5 mmol (≈232 mg)/h are recommended [28, 29].

Further noteworthy is the refeeding syndrome, occurring in patients where nutrition is reinstated, which can present itself with life-threatening hypophosphatemia if not detected and supplemented [4]. Generally, a risk assessment to develop a refeeding syndrome is performed, which varies but generally includes the current and previous weight, height, feeding habits, weight kinetics, and risky clinical conditions, e.g., anorexia nervosa or alcohol use disorder [4]. Guidelines agree on baseline phosphate testing before nutrition initiation but differ in terms of when to monitor and when and how to supplement [4, 19, 29, 34]. Initial monitoring is most often recommended daily, with longer intervals over time but shorter intervals in higher-risk patients. While in most other forms of acute hypophosphatemia, supplementation is most commonly recommended if hypophosphatemia is severe or moderate and symptomatic [8, 28], it is reasonable to supplement phosphate at higher levels in patients at risk of refeeding to avoid the development of symptomatic hypophosphatemia, but it is unclear at which phosphate level. One suggestion is when the phosphate concentration is < 0.8 mmol/L (2.5 mg/dL) [34]. Additionally, enteral and parenteral nutrition preparations already contain phosphate, ranging from 700 to 2000 mg (≈22.6–38.7 mmol)/L [29].

In summary, the most common causes of acute hypophosphatemia are transient and rarely need treatment, except for severe or moderate symptomatic hypophosphatemia. In patients at risk of refeeding syndrome and nutrition initiation, supplementation is warranted at higher phosphate levels to prevent symptomatic hypophosphatemia.

Several drugs can cause hypophosphatemia, most commonly through renal phosphate loss. Notably, the iron infusion preparation FCM has been shown to cause hypophosphatemia, ranging from acute life-threatening symptoms to osteomalacia [8, 38]. FCM leads to a 3–sixfold increase in FGF-23, which in turn leads to an increase in urinary phosphate excretion, thus potentially causing acute hypophosphatemia [55]. Especially with repeat infusions and undetected hypophosphatemia, the risk for chronic hypophosphatemia and, accordingly, osteomalacia increases [36]. It is currently unknown why other iron preparations do not lead to the same extent of increase in FGF-23, although it is important to note that other iron infusions such as ferric derisomaltose and ferrumoxytol can also lead to hypophosphatemia, albeit less commonly and less severely [8, 36, 38]. In the included works, recommendations for phosphate testing differ from universal screening before FCM infusions to screening in those with repeat infusions and those with musculoskeletal symptoms and ongoing fatigue after infusion [8, 36, 38]. Due to the described data and recent emerging data from clinical trials [56, 57], the U.S. Food and Drug Administration (FDA) has adjusted its warning label for FCM infusions in November 2024 to recommend testing for hypophosphatemia in patients at risk for low serum phosphate who require a repeat course of treatment or for any patient who receives a repeat course of treatment within 3 months [58]. Risk factors for low serum phosphate include a history of gastrointestinal disorders associated with malabsorption of fat-soluble vitamins or phosphate, concurrent or prior use of medications that affect proximal renal tubular function, hyperparathyroidism, vitamin D deficiency, and malnutrition [58]. The European Medicines Agency (EMA) also includes a warning on hypophosphatemic osteomalacia in their Summary of Product Characteristics for FMC infusions, stating the need for phosphate testing in patients with multiple administrations at higher doses or long-term treatment, and those with existing risk factors for hypophosphatemia, as well as patients with worsening fatigue, myalgias, or bone pain [59]. Since elevated FGF-23 underlies the disorder, phosphate supplementation is not expected to sustainably correct hypophosphatemia, thus the most common treatment recommendation was to discontinue or, if not possible, switch iron preparations [8, 38]. TDF, which is used to treat hepatitis B and HIV, can cause tubular kidney dysfunction and thus lead to hypophosphatemia, which is why older reviews recommend regular phosphate testing for all patients treated with TDF [30, 60, 61]. However, studies, mainly in patients with HIV, have shown that it is a rare complication, and in newer HIV guidelines, testing is recommended only in the case of certain prompts, such as preexisting CKD, worsening GFR, or proteinuria [21, 22]. These recommendations can probably be used for patients with hepatitis B as well. Notably, these conditions disproportionately affect low-income countries, where resources can influence monitoring strategies. Other drugs known to cause hypophosphatemia include mTOR inhibitors [35], aluminum-based antacids (if taken in large quantities over a longer period of time, as they bind to phosphate in the gastrointestinal tract) [62], tyrosine kinase inhibitors (particularly imatinib) [63, 64] and vascular endothelial growth factor inhibitors [65]. However, we found no recommendations on when to evaluate for hypophosphatemia in these medications.

In summary, if clinicians are aware of the drugs that cause hypophosphatemia, symptoms of either chronic or acute hypophosphatemia should prompt phosphate testing. Additionally, phosphate screening should be performed in patients with repeat FCM infusions.

While end-stage CKD is associated mainly with hyperphosphatemia, there are two main instances where hypophosphatemia occurs: first, in intensive dialysis, with the solution being to use phosphate-enriched hemodialysates [20, 40, 66]. Second, in the immediate post-kidney transplant stage [16, 25, 37], persistently high levels of FGF-23 and parathyroid hormone with significantly improved GFR lead to renal phosphate loss [37]. Most patients develop hypophosphatemia within the first 3 months after kidney transplantation, and while it is most often self-limited, hypophosphatemia can sometimes persist for months [67]. The KDIGO guidelines thus recommend weekly phosphate monitoring in the immediate post-transplant stage until stabilization, but it is unclear when phosphate should be supplemented [16]. As these conditions are most often self-limited, a somewhat lower threshold seems reasonable, with suggestions ranging from 0.5–0.65 mmol/L (1.5–2 mg/dL) to < 1 mmol/L (3.1 mg/dL) in cases of muscle weakness [25, 37].

This review has several limitations. First, we excluded non-English publications and studies from intensive care settings. These decisions were made to ensure consistent interpretation and applicability across general clinical contexts, as ICU protocols and language barriers may introduce substantial heterogeneity and complexity. Second, many of the included sources rely heavily on expert opinion rather than systematic evidence synthesis, which may introduce bias and limit generalizability. Third, the possibility of publication bias cannot be excluded, particularly given the tendency to publish guidelines and reviews with positive or actionable findings. Finally, while we aimed to include the most recent and comprehensive sources with dual independent screening, the variability in format and scope across publications may have led to the omission of relevant but less prominent recommendations.

## Conclusions

In conclusion, data quality for the measurement and supplementation of phosphate in the context of hypophosphatemia or suspicion of hypophosphatemia varies, but overall, chronic hypophosphatemia testing should be performed in patients with ongoing unclear musculoskeletal complaints, especially if drugs known to cause hypophosphatemia are taken or during osteoporosis evaluation. Among drug-related causes, FMC–associated hypophosphatemia warrants particular attention; clinicians should monitor phosphate around repeat infusions and manage cases individually given ongoing uncertainties about optimal dosing and monitoring. Supplementation for chronic hypophosphatemia overall should be given to symptomatic individuals if the causative agent cannot be eliminated by the oral route via multiple doses. The goal should most likely be clinical improvement, and doses and monitoring remain unclear. For acute hypophosphatemia, it is important to know potential causes, such as treatment of alcoholic ketoacidosis or refeeding syndrome, and potential signs, such as arrhythmia or respiratory distress, to prompt phosphate testing. It is unclear when to treat it, especially as most causes of acute hypophosphatemia are self-limited, but the general consensus seems to be to treat severe or moderate symptomatic hypophosphatemia, with differing recommendations for dosing and monitoring. More studies are needed on when to evaluate acute hypophosphatemia and how to treat and monitor acute and chronic hypophosphatemia.

## Supplementary Information

Additional file 1. Table S1-S2. Table S1-Quality assessment via the AGREE II tool. Table S2-Detailed search strategy.

## Data Availability

The datasets used and/or analysed during the current study are available from the corresponding author on reasonable request. The detailed search strategy can be found in Additional file 1: Table S2.

## Abbreviations

FGF-23: Fibroblast growth factor 23
NICE: National Institute of Health and Care Excellence
ICU: Intensive care unit
AGREE: Appraisal of Guidelines, Research and Evaluation
DXA: Dual x-ray absorptiometry
COPD: Chronic obstructive pulmonary disease
CKD: Chronic kidney disease
BMI: Body mass index
FCM: Ferric carboxymaltose
TDF: Tenofovir disoproxil fumarate
mTOR: Mechanistic target of rapamycin
HIV: Human immunodeficiency virus
KDIGO: Kidney Disease: Improving Global Outcomes
ASPEN: America Society for Parenteral and Enteral Nutrition
EACS: European AIDS Clinical Society
OARAC: Office of AIDS Research Advisory Council
HHS: Hyperglycemic hyperosmolar syndrome
HBV: Hepatitis B virus
eGFR: Estimated glomerular filtration rate
TIO: Tumor-induced osteomalacia
i.v.: Intravenous
PTH: Parathyroid hormone
