# Supplementary material for: Indications for the evaluation and supplementation of hypophosphatemia: an umbrella systematic review of reviews and guidelines
Source: BMC Med. 2025 Oct 27;23:591. doi: 10.1186/s12916-025-04415-1 (PMC12560374; doi:10.1186/s12916-025-04415-1)
Supplement: Supplementary file 1 — Additional file 1. Table S1-S2. Table S1-Quality assessment via the AGREE II tool. Table S2-Detailed search strategy. [file 12916_2025_4415_MOESM1_ESM.docx]

**Supplementary Table S1:** Quality assessment via the AGREE II tool

| Author/Year | Fukumoto 2015(7) | Ackah 2022(21) | Gregson 2022(15) | Jan de Beur 2023(12) | Haffner 2019(11) | Trombetti 2022(40) | Cherian 2024(25) | Javaid 2019(13) | Aljuraibah 2022(22) | Florenzano 2020(26) | Bilezikian 2022(16) | Fukumoto 2008(41) | ASPEN 2020(3) | Reber 2019(33) | Kraft 2015(28) | NICE 2017(17) | Gosmanov 2014(27) | Milionis 2005(31) | Long 2021(30) | Weber 2019(38) |
| --- | --- | --- | --- | --- | --- | --- | --- | --- | --- | --- | --- | --- | --- | --- | --- | --- | --- | --- | --- | --- |
| Domain 1: scope and purpose | | | | | | | | | | | | | | | | | | | | |
| Objective |  |  |  |  |  |  |  |  |  |  |  |  |  |  |  |  |  |  |  |  |
| Health questions |  |  |  |  |  |  |  |  |  |  |  |  |  |  |  |  |  |  |  |  |
| Population |  |  |  |  |  |  |  |  |  |  |  |  |  |  |  |  |  |  |  |  |
| Domain 2: stakeholder involvement | | | | | | | | | | | | | | | | | | | | |
| Professional groups |  |  |  |  |  |  |  |  |  |  |  |  |  |  |  |  |  |  |  |  |
| Views of target |  |  |  |  |  |  |  |  |  |  |  |  |  |  |  |  |  |  |  |  |
| Users |  |  |  |  |  |  |  |  |  |  |  |  |  |  |  |  |  |  |  |  |
| Domain 3: rigour of development | | | | | | | | | | | | | | | | | | | | |
| Systematic methods |  |  |  |  |  |  |  |  |  |  |  |  |  |  |  |  |  |  |  |  |
| Selection process |  |  |  |  |  |  |  |  |  |  |  |  |  |  |  |  |  |  |  |  |
| Limitations |  |  |  |  |  |  |  |  |  |  |  |  |  |  |  |  |  |  |  |  |
| Recommendation methods |  |  |  |  |  |  |  |  |  |  |  |  |  |  |  |  |  |  |  |  |
| Benefits and risks |  |  |  |  |  |  |  |  |  |  |  |  |  |  |  |  |  |  |  |  |
| Link |  |  |  |  |  |  |  |  |  |  |  |  |  |  |  |  |  |  |  |  |
| External review |  |  |  |  |  |  |  |  |  |  |  |  |  |  |  |  |  |  |  |  |
| Update |  |  |  |  |  |  |  |  |  |  |  |  |  |  |  |  |  |  |  |  |
| Domain 4: clarity of presentation | | | | | | | | | | | | | | | | | | | | |
| Ambiguity |  |  |  |  |  |  |  |  |  |  |  |  |  |  |  |  |  |  |  |  |
| Options |  |  |  |  |  |  |  |  |  |  |  |  |  |  |  |  |  |  |  |  |
| Key recommendations |  |  |  |  |  |  |  |  |  |  |  |  |  |  |  |  |  |  |  |  |
| Domain 5: applicability | | | | | | | | | | | | | | | | | | | | |
| Facilitators and barriers |  |  |  |  |  |  |  |  |  |  |  |  |  |  |  |  |  |  |  |  |
| Tools |  |  |  |  |  |  |  |  |  |  |  |  |  |  |  |  |  |  |  |  |
| Resource implications |  |  |  |  |  |  |  |  |  |  |  |  |  |  |  |  |  |  |  |  |
| Monitoring |  |  |  |  |  |  |  |  |  |  |  |  |  |  |  |  |  |  |  |  |
| Domain 6: editorial independence | | | | | | | | | | | | | | | | | | | | |
| Funding |  |  |  |  |  |  |  |  |  |  |  |  |  |  |  |  |  |  |  |  |
| Competing interests |  |  |  |  |  |  |  |  |  |  |  |  |  |  |  |  |  |  |  |  |

The AGREEII tool is rated from 1--7, with 1 indicating the lowest score and 7 indicating the highest score. For easier overview, we decided on 3 categories: 1-2 red dots, meaning the item was not or very poorly addressed (Did they address this item satisfactorily or at all? No.); 3-5: yellow dots, meaning that the item was partially addressed (partial yes. ); 6-7: green dots, meaning the item was addressed (Yes. ).

| Author/Year | Miller 2000(32) | Boots 2022(24) | Schaefer 2022(35) | Vilaca 2022(37) | Rodriguez 2010(34) | Lampertico 2016(29) | EACS 2023(19) | OARAC 2024(20) | KDIGO 2017(14) | Vangala 2018(36) | Baia 2015(23) | Nesrallah 2013(18) | Sam 2006(39) |
| --- | --- | --- | --- | --- | --- | --- | --- | --- | --- | --- | --- | --- | --- |
| Domain 1: scope and purpose | | | | | | | | | | | | | |
| Objective |  |  |  |  |  |  |  |  |  |  |  |  |  |
| Health questions |  |  |  |  |  |  |  |  |  |  |  |  |  |
| Population |  |  |  |  |  |  |  |  |  |  |  |  |  |
| Domain 2: stakeholder involvement | | | | | | | | | | | | | |
| Professional groups |  |  |  |  |  |  |  |  |  |  |  |  |  |
| Views of target |  |  |  |  |  |  |  |  |  |  |  |  |  |
| Users |  |  |  |  |  |  |  |  |  |  |  |  |  |
| Domain 3: rigour of development | | | | | | | | | | | | | |
| Systematic methods |  |  |  |  |  |  |  |  |  |  |  |  |  |
| Selection process |  |  |  |  |  |  |  |  |  |  |  |  |  |
| Limitations |  |  |  |  |  |  |  |  |  |  |  |  |  |
| Recommendation methods |  |  |  |  |  |  |  |  |  |  |  |  |  |
| Benefits and risks |  |  |  |  |  |  |  |  |  |  |  |  |  |
| Link |  |  |  |  |  |  |  |  |  |  |  |  |  |
| External review |  |  |  |  |  |  |  |  |  |  |  |  |  |
| Update |  |  |  |  |  |  |  |  |  |  |  |  |  |
| Domain 4: clarity of presentation | | | | | | | | | | | | | |
| Ambiguity |  |  |  |  |  |  |  |  |  |  |  |  |  |
| Options |  |  |  |  |  |  |  |  |  |  |  |  |  |
| Key recommendations |  |  |  |  |  |  |  |  |  |  |  |  |  |
| Domain 5: applicability | | | | | | | | | | | | | |
| Facilitators and barriers |  |  |  |  |  |  |  |  |  |  |  |  |  |
| Tools |  |  |  |  |  |  |  |  |  |  |  |  |  |
| Resource implications |  |  |  |  |  |  |  |  |  |  |  |  |  |
| Monitoring |  |  |  |  |  |  |  |  |  |  |  |  |  |
| Domain 6: editorial independence | | | | | | | | | | | | | |
| Funding |  |  |  |  |  |  |  |  |  |  |  |  |  |
| Competing interests |  |  |  |  |  |  |  |  |  |  |  |  |  |

The AGREEII tool is rated from 1 (strongly disagree) to 7 (strongly agree). For easier overview, we decided on 3 categories: 1-2 red dots, meaning the item was not or very poorly addressed (Did they address this item satisfactorily or at all? No.); 3-5: yellow dots, meaning that the item was partially addressed (partial yes. ); 6-7: green dots, meaning the item was addressed (Yes. )

**Supplementary Table S2:** Detailed search strategy

| Database | Search Strategy |
| --- | --- |
| MEDLINE ALL (Ovid) | Ovid MEDLINE(R) ALL <1946 to now>  1 exp Hypophosphatemia/ 4045 2 (hypophosphatemi* or hypo-phosphatemi* or phosphaturia? or "vitamin d resistant rickets" or hypophosphataemi* or hypo-phosphataemi* or hypophosphoremi* or hypo-phosphoremi* or hyperphosphaturia? or hyper-phosphaturia?).ti,ab. 7480 3 1 or 2 8405 4 Phosphorus/ or Phosphates/ or phosphoric acids/ or calcium phosphates/ or calcium pyrophosphate/ 130315 5 (Phosphate? or "phosphoric acid?" or phosphorus or "calcium phosphate?" or "calcium pyrophosphate?" or "calcium diphosphate?" or "calcium pyrophosphate?").ti,ab. 352363 6 4 or 5 400859 7 diagnosis/ or diagnosis, differential/ or clinical laboratory techniques/ or diagnostic tests, routine/ or "direct-to-consumer screening and testing"/ or physical examination/ or early diagnosis/ or incidental findings/ or diagnostic errors/ or "diagnostic techniques and procedures"/ or Delayed Diagnosis/ or exp Clinical Decision-Making/ 649323 8 (diagnos* or screen* or test* or examination).ti,ab. 6981434 9 7 or 8 7284998 10 3 and 6 and 9 1368 11 Dietary Supplements/ or Therapeutics/ or Phosphorus, Dietary/ 83340 12 (diet* or supplement* or replacement therap* or substitution therap* or therap* or treat*).ti,ab. or (tu or th or dh or dt).fs. 10424868 13 11 or 12 10434531 14 3 and 6 and 13 3071 15 10 or 14 3500 16 exp Child/ or Adolescent/ or (child or children or infant? or adolescent?).ti. 3594261 17 exp adult/ or ((child$ and adult$) or (adolescent$ and adult$)).ti. 7869956 18 15 not (16 not 17) 2937 19 (exp animals/ or animal experimentation/ or models, animal/ or exp plants/ or exp fungi/) not humans/ 5484616 20 18 not 19 2426 21 (ICU? or Intensive Care Unit? or SICU? or PICU?).ti. 46864 22 20 not 21 2405 23 limit 22 to english language 2082 |
| Embase (Ovid) | Embase <1974 to now>  1 hypophosphatemia/ or hypophosphatemic rickets/ 13530 2 (hypophosphatemi* or hypo-phosphatemi* or phosphaturia? or "vitamin d resistant rickets" or hypophosphataemi* or hypo-phosphataemi* or hypophosphoremi* or hypo-phosphoremi* or hyperphosphaturia? or hyper-phosphaturia?).ti,ab. 10929 3 1 or 2 17402 4 phosphorus/ or phosphate/ or phosphoric acid/ or calcium phosphate/ or calcium pyrophosphate/ 200452 5 (Phosphate? or "phosphoric acid?" or phosphorus or "calcium phosphate?" or "calcium pyrophosphate?" or "calcium diphosphate?" or "calcium pyrophosphate?").ti,ab. 391977 6 4 or 5 455024 7 diagnosis/ or diagnostic procedure/ or delayed diagnosis/ or differential diagnosis/ or early diagnosis/ or incidental finding/ or laboratory test/ or laboratory technique/ or physical examination/ or diagnostic error/ or missed diagnosis/ or clinical decision making/ 2381595 8 (diagnos* or screen* or test* or examination).ti,ab. 9457689 9 7 or 8 10351571 10 3 and 6 and 9 3072 11 dietary supplement/ or therapy/ or time to treatment/ or treatment indication/ or phosphate intake/ or diet supplementation/ or mineral supplementation/ 1518533 12 (diet* or supplement* or replacement therap* or substitution therap* or therap* or treat*).ti,ab. or (dt or th).fs. 13519260 13 11 or 12 14074018 14 3 and 6 and 13 5719 15 10 or 14 6402 16 exp child/ or exp adolescent/ or (child or children or infant? or adolescent?).ti. 3917248 17 exp adult/ or ((child$ and adult$) or (adolescent$ and adult$)).ti. 10122479 18 15 not (16 not 17) 5208 19 (exp animal/ or exp invertebrate/ or nonhuman/ or animal experiment/ or animal tissue/ or animal model/ or exp plant/ or exp fungus/) not (exp human/ or human tissue/) 7489229 20 18 not 19 4507 21 (ICU? or Intensive Care Unit? or SICU? or PICU?).ti. 72411 22 20 not 21 4458 23 limit 22 to english 4060 |
| Cochrane Library (Wiley) | #1 (hypophosphatemi* or hypo-phosphatemi* or phosphaturia? or "vitamin d resistant rickets" or hypophosphataemi* or hypo-phosphataemi* or hypophosphoremi* or hypo-phosphoremi* or hyperphosphaturia? or hyper-phosphaturia?):ti,ab 536 #2 (Phosphate? or (phosphoric NEXT acid?) or phosphorus or (calcium NEXT phosphate?) or (calcium NEXT pyrophosphate?) or (calcium NEXT diphosphate?) or (calcium NEXT pyrophosphate?)):ti,ab 11741 #3 (diagnos* or screen* or test* or examination):ti,ab 583469 #4 #1 AND #2 AND #3 75 #5 (diet* or supplement* or (replacement NEXT therap*) or (substitution NEXT therap*) or therap* or treat*):ti,ab 1093784 #6 #1 AND #2 AND #5 241 #7 #4 OR #6 246 #8 (child or children or infant? or adolescent?):ti 108225 #9 ((child? and adult?) or (adolescent? and adult?)):ti 3240 #10 #7 NOT (#8 NOT #9) 201 #11 (ICU? or (Intensive NEXT Care NEXT Unit?) or SICU? or PICU?):ti 5052 #12 #10 NOT #11 197 |
| Google Scholar (via Publish or Perish) | hypophosphatemia\|hypophosphataemia\| phosphate\|phosphates diagnosis\|diagnostic\|screening\|therapy\|supplementation (Limited to 200 records) |
| NICE | Search term: hypophosphataemia |
